# Isolation and Characterization of Natural Nanoparticles in Naoluo Xintong Decoction and Their Brain Protection Research

**DOI:** 10.3390/molecules27051511

**Published:** 2022-02-23

**Authors:** Guodong Zhao, Lu Hong, Mingming Liu, Huihui Jiang, Daiyin Peng, Ling He, Weidong Chen

**Affiliations:** 1 School of Pharmacy, Anhui University of Chinese Medicine, Hefei 230012, China; 18712283909@163.com (G.Z.); youxiaoyuqu@163.com (L.H.); 18055873835@163.com (M.L.); jhh121389@163.com (H.J.); 2 Key Laboratory of Xin’an Medicine Ministry of Education, Anhui University of Chinese Medicine, Hefei 230038, China; pengdy@ahtcm.edu.cn

**Keywords:** ischemic stroke, naoluo xintong, nanoparticles, brain protection

## Abstract

Currently, researchers use modern analytical techniques in a unique perspective of physical pharmacy to analyze the phase composition of traditional Chinese medicine (TCM) and have discovered that natural nanoparticles commonly exist in decoctions. This study aims to isolate and characterize the structure and composition of nanoparticles in Naoluo Xintong (NLXT) and investigate whether the brain protection effect of NLXT is closely related to NLXT-Nanoparticles (NLXT-NPs). Firstly, the dialysis-centrifugation method was used to separate the nanoparticles and then their size distribution, potential, and morphology were characterized. In addition, infrared spectroscopy and ultra-high performance liquid chromatography-quadrupole-time of flight-mass spectrometer (UPLC-Q-TOF-MS) technology were used to analyze the composition of nanoparticles. As for the pharmacodynamic experiment, Sprague Dawley (SD) rats were randomly divided into sham, Middle cerebral artery occlusion (MCAO) model, NLXT, NLXT with nanoparticles removing (NLXT-RN), NLXT-RN+Nanoparticles (NLXT-RN+NPs), and NLXT-NPs groups. After administration, the neurological function, histopathological changes, oxidative stress, and apoptosis level were measured. Our research showed that NLXT-NPs are mainly composed of polysaccharides, proteins, and saponins, with typical characteristics of two hundred-nanometer size and negatively loaded. NLXT can improve nerve function, reduce oxidative stress, and inhibit cell apoptosis. However, removing nanoparticles can significantly reduce the brain-protective effect of NLXT, which indicates that NLXT-NPs play an essential role in the efficacy of NLXT.

## 1. Introduction

Stroke is one of the three high mortality diseases in China. According to a report, about 2.3 million people died of this disease in 2020 [1]. Ischemic stroke (IS) is the main form of stroke, characterized by high disability and recurrence [2]. The clinical treatment of IS primarily uses recombinant-tissue plasminogen activator (r-tPA) drugs for intravenous thrombolysis [3]. However, this method is often accompanied by bleeding risks; more importantly, intravenous thrombolysis has strict indications and contraindications, and most patients do not meet these standards. On the contrary, as a natural product, traditional Chinese medicines (TCM) such as *Astragali radix* and *Notoginseng radix* generally have the dual effects of curing diseases and health care [4,5]; further developed preparations of corresponding TCM often show high biocompatibility [6]. TCM has been used in treating stroke for over a thousand years and generally has the characteristics of multi-component and multi-target action [7,8]. Using modern analytical methods to clarify the material basis and efficiency mechanism is the focus of TCM research [9,10].

The decoction is one of the most ordinarily used clinical dosage forms in TCM [11]. Bioactive ingredients in TCM decoction (TCMD) are tied for the therapeutic effects. Previous research on the pharmacodynamic material basis of TCMD focuses more on small molecule active ingredients, and the research object is often the macro-integration [12,13]. At present, more and more researchers discovered that nano-scale particles such as nanoparticles, micelle and lipid commonly exist in decoctions. The formation of these nano-scale particles is closely related to the pharmacological effects of decoctions [14,15]. For example, polysaccharide nanoparticles in the Coptis Chinensis decoction can encapsulate active ingredients and produce solubilization and absorption promotion to enhance the curative effect [16]. In the process of aconite licorice decoction, glycyrrhizin (a natural surfactant) can be self-assembled into micelle particles and encapsulate toxic components, delaying or inhibiting their absorption and reducing toxic side effects [17]. Moreover, ginger-derived lipid particles can target the delivery of siRNA for the treatment of colitis and has a synergistic anti-inflammatory effect [14]. In conclusion, TCM-derived nano-scale particles have lower cytotoxicity and possess attenuating and synergistic effects. Research on decoction nano-scale particles has attracted more and more attention. These studies mainly revolve around chemical composition, structure characterization [18,19], formation mechanism [20], improving the bioavailability of co-existing compounds, and their pharmaceutics application [21]. Clarifying the role of nano-scale particles in decoction is of great innovative significance for revealing the material basis of TCMD.

NLXT is a clinically proven prescription used by Xin’an Medicine to treat ischemic stroke [22]. It consists of six herbs and one animal medicine: *Astragali radix*, *Chuanxiong rhizome*, *Noto-ginseng radix et rhizome*, *Gastrodiae rhizome*, *Chilopoda Scolopendra*, *Carthami Flos*, and *Angelicae Sinensis Radix* in the ratio of 15:5:3:5:2:5:5. The prescription relies on Astragalus as the monarch medicine, which played a fundamental role in the pharmacological effect of NLXT. Chuanxiong and Panax Notoginseng act as ministerial medicines; Gastrodiae rhizome and Scolopendra play an assistant role due to their function in regulating meridians; Carthami Flos and Angelicae Sinensis radix are regarded as “guide herbs” with their harmonious function [23,24]. Previous studies reported that NLXT could improve brain blood circulation, anti-apoptosis, pro-angiogenesis, and nerve regeneration [25,26]. The small molecule active ingredients in NLXT decoction play a crucial role in treating IS and have been explored previously [27]. However, the active components of the TCMD are not only small and molecular; the presence of nanoparticles in the decoction can also show certain pharmacological activities. In this study, we found that there are nano-scale particles with a size distribution of 200 nm~400 nm that existed in the NLXT decoction after dialysis-centrifugation. In order to characterize the structure and composition of NLXT-NPs and investigate whether the brain protection effect of NLXT is related to NLXT-NPs, this study constructed an MCAO rat model to simulate an ischemic stroke. After administration with NLXT, NLXT-RN, NLXT-RN+NPs, and NLXT-NPs, the neurological function score (NFS), cerebral infarction volume, histopathological changes, oxidative stress, and apoptosis level were measured and analyzed.

## 2. Results

### 2.1. The Particle Size, Potential, and Morphology of NLXT-NPs

**Figure 1:**
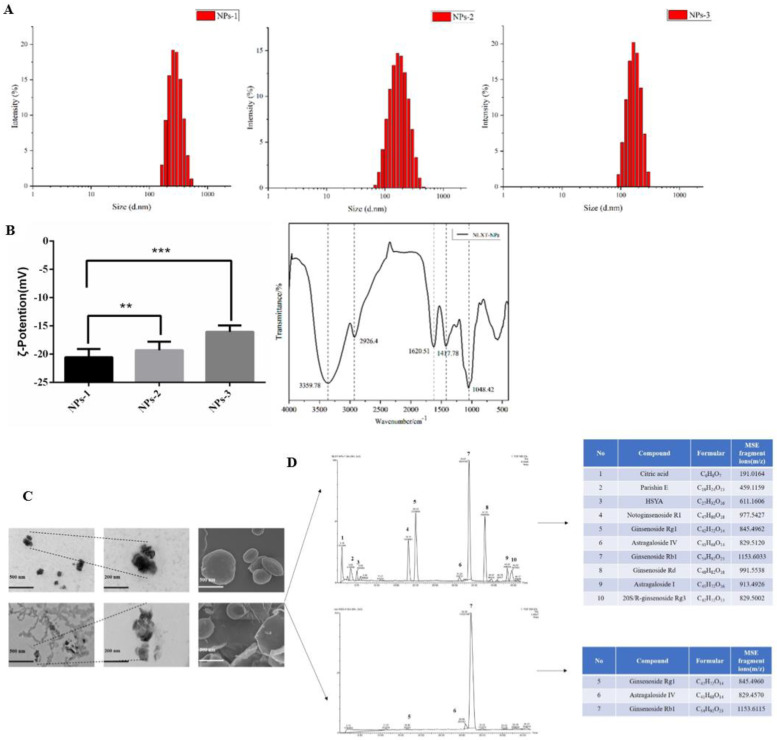
(**A**) The particle size distribution diagram of nanoparticles with different concentrations (NPs−1 3.80 mg·mL^−1^, NPs−2 1.90 mg·mL^−1^ and NPs–3 0.95 mg·mL^−1^); (**B**) Representation of the potential of nanoparticle solutions with different concentrations, values are the mean± SD (*n* = 6), and the infrared spectra of NLXT-NPs; Compared with NPs-1, ** *p* < 0.05, *** *p* < 0.01; (**C**) The TEM and SEM picture of NLXT-NPs dialysis in 10 h (up) and 12 h (down); (**D**) The chemical composition of NLXT-NPs dialysis in 10 h (up) and 12 h (down).

**Figure 2:**
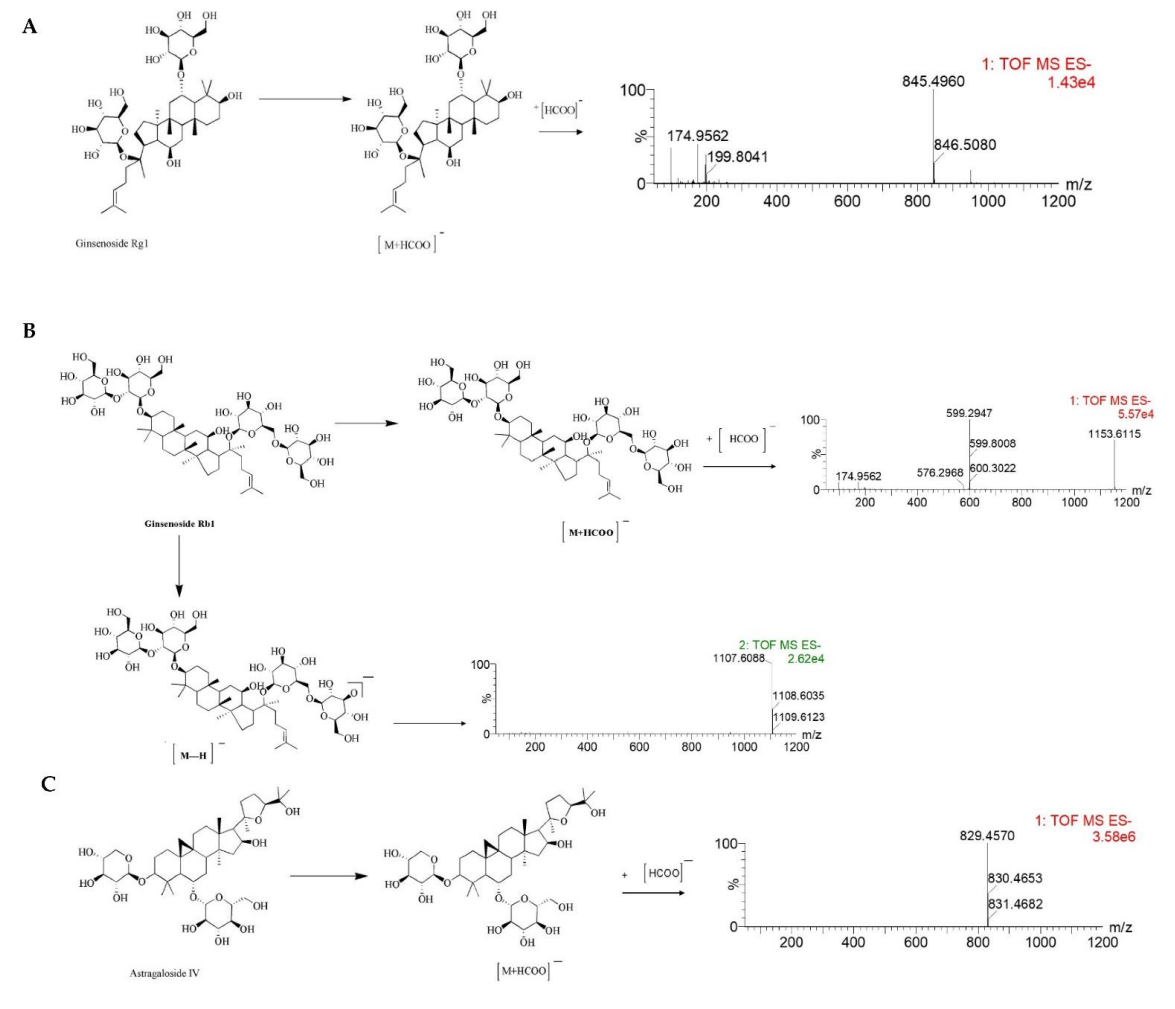
The MS/MS spectrum and possible fragmentation pathway of Ginsenoside Rg1 (**A**), Ginsenoside Rb1 (**B**), and Astragaloside IV (**C**).

In this research, we chose a gentle way to separate the nanoparticles: high-speed centrifugation combined with dialysis, which can better maintain the natural state of nanoparticles. In our research, the size distribution and potential of nanoparticles were closely related to the solution concentration. The particle size and potential of the NLXT-NPs solution at 0.95, 1.90, and 3.80 mg·mL^−1^ are respectively (185.50 ± 18.93 nm, −16.07 ± 0.46 mV), (239.80 ± 19.44 nm, −19.33 ± 0.62 mV), and (353.13 ± 21.12 nm, −20.57± 0.60 mV). The infrared absorption spectrum of the nanoparticles showed a remarkable characteristic of polysaccharides. As shown in Figure 1B, the absorption peak of NLXT-NPs at 3359.7 cm^−1^ belongs to the stretching vibration of -OH, and the peak at 2926.4 cm^−1^ is due to the stretching vibration of γ-CH2 and C-H. Furthermore, the peak of 1620.5 cm^−1^ is attributed to the vibration of the C-O bond, and the peaks of 1417.7 cm^−1^ belong to the bending vibration of δ-CH2. The above results suggest that the majority components of NLXT-NPs may be polysaccharides. Therefore, we used a phenol-sulfuric acid method and the Coomassie brilliant blue method [28] to determine the polysaccharide and protein content of the nanoparticles. The results show that the polysaccharide content in the nanoparticles is 82.75% ± 2.16%, and the protein content is 6.20% ± 0.14%. Besides, TEM and SEM were used to observe the morphology of nanoparticles, and it was found that NLXT-NPs had uneven particle size distribution, relatively regular shapes, and were mostly clustered. It is worth mentioning that the morphology of the nanoparticles obtained after dialysis for 10 h and 12 h was significantly different. As shown in Figure 2C, the morphology of the particles after dialysis for 12 h can be observed to be broken and showed loss of particle integrity, which indicates that long-term dialysis may destroy the aggregate state of nanoparticles. Therefore, in this study, the separation of nanoparticles was performed by dialysis for 10 h.

### 2.2. Chemical Composition of NLXT-NPs

UPLC-Q-TOF-MS has proven to be a powerful technique for qualitative analysis of multi-components in a complex system due to its satisfactory UPLC separation efficiency and powerful structural characterization of Q-TOF-MS. According to the result of UPLC-Q-TOF-MS, ten chemical components were detected and determined by UNIFI and Masslynx 4.1 based on the accurate mass, fragment ions, neutral losses, mass error, reference substance, isotope information, the intensity of fragments, and retention time. The ten components were mostly Astragaloside and Notoginsenoside, besides Citric acid, Parishin E, and Hydroxysafflor yellow A can also be determined. At the same time, we analyzed the chemical composition of the nanoparticles obtained by dialysis for 12 h, and only three active ingredients were detected. This result may relate to the precipitation of the active ingredients caused by the depolymerization of the nanoparticles.

Furthermore, the MS/MS stands for secondary mass spectrometry, which can fragment the ions detected by the first mass spectrometry and then perform the secondary mass spectrometry detection to more accurately characterize the compound. In this study, the Ginsenoside Rg1, Ginsenoside Rb1, and Astragaloside IV were used as examples to illustrate the cleavage pattern of saponins and Astragaloside in NLXT-NPs. As shown in Figure 2, Ginsenoside Rg1 displayed the parent ion at low collision energy at *m*/*z* 845.4960 [M+HCOO]^−^. Ginsenoside Rb1 displayed the parent ion at low collision energy at m/z 1153.6115 [M+HCOO]^−^, and the major fragment ions in the secondary mass spectrum at *m*/*z* 1107.6088 [M-H]^−^. Astragaloside IV displayed parent ion at low collision energy at *m*/*z* 829.4570 [M+HCOO]^−^. These results were consistent with previous research that [29] the precursor ions of the saponins and Astragaloside exist mainly as [M+HCOO]^−^. Therefore, combined with previous research and structural screening, we can make further identification of the compounds in NLXT-NPs.

### 2.3. NLXT-NPs Can Improve the Neural Protective Effects of NLXT on MCAO Rats

As shown in Table 1, the NFS of modeling rats was notably decreased compared with the sham group (*p* < 0.05). It was gradually restored following treatment with NLXT-NPs (9.33 ± 1.86), NLXT-RN (10.17 ± 1.17), NLXT-RN+NPs (12.83 ± 0.75), and NLXT (13.17 ± 1.17). This finding suggested that NLXT could promote the recovery of neurological function in MCAO rats; however, removing NLXT-NPs may significantly reduce its protective effect (*p* < 0.05). Interestingly, the impact of NLXT-RN+NPs showed no significant difference compared with the NLXT group (*p* > 0.05), which indicates that NLXT-NPs may have a non-negligible function on the neuroprotection effect of NLXT. Furthermore, the results of cerebral infarction volume assessment showed a similar trend. There was no infarction in the sham group, and the cerebral infarction volume in the MCAO group was the highest (47.83 ± 5.31)%. Compared with the model group, the NLXT group could significantly reduce the cerebral infarction volume (*p* < 0.05). Importantly, there was no significant difference between NLXT and NLXT-RN+NPs (*p* > 0.05), but the removal of nanoparticles will reduce the protective impact of NLXT (*p* < 0.05). Moreover, compared with the MCAO group, NLXT-NPs also significantly reduced infarct volume (*p* < 0.05).

### 2.4. Pathological Changes of Ischemic Brain Tissue

**Figure 3:**
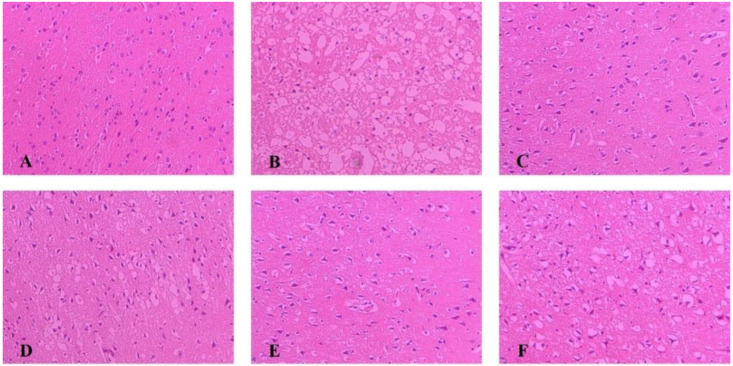
Histopathological changes in the cerebral cortex of rats in each group were observed by HE staining (×200). (**A**) Sham group; (**B**) MCAO group; (**C**) NLXT-group; (**D**) NLXT-RN group; (**E**) NLXT-RN+NPs group; (**F**) NLXT-NPs group.

As shown in Figure 3, The morphology of neurons in the sham group was normal, with dense tissues, neatly arranged cells, and the nucleus located in the center of the cells; after modeling, brain tissue appeared loose and with noticeable swelling, reduced cell numbers, disordered cell arrangement, and constriction of the neuronal nucleus. Each administration group can reduce brain tissue damage caused by MCAO. Among them, the improvement degree of NLXT and NLXT-RN+NPs groups were the most obvious; the brain tissue morphology is similar to the sham group. Although NLXT-NPs and NLXT-RN showed a certain improvement effect, there is still a gap compared with the NLXT and NLXT-RN+NPs groups.

### 2.5. Oxidative Stress Level Regulation

**Figure 4:**
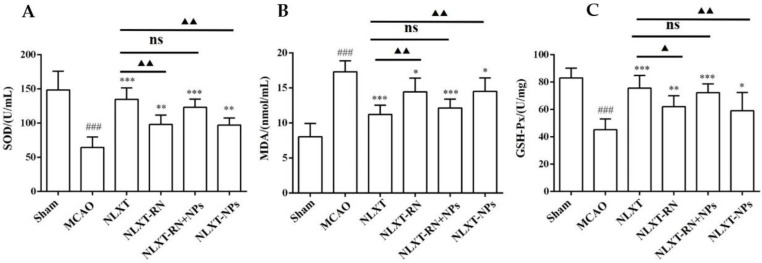
Effect of administration groups on the level of SOD (**A**), GSH-Px (**B**), and MDA (**C**) of MCAO rat models (*n* = 8). ^###^ *p* < 0.001, compared with sham group; * *p* < 0.05, ** *p* < 0.01, *** *p* < 0.001, compared with MCAO group; ns: no significant difference, ^▲^ *p* < 0.05, ^▲▲^ *p* < 0.01, compared with NLXT group.

As shown in Figure 4, after modeling, the enzyme activity of superoxide dismutase (SOD) and glutathione peroxidase (GSH-PX) decreased obviously (*p* < 0.001), and the malondialdehyde (MDA) level increased (*p* < 0.001), compared with the sham group. In addition, each administration group could improve the scope of SOD and GSH-Px and reduce the content of MDA. Among these, the effect of the NLXT group was the best one (*p* < 0.01), and there was no significant difference between NLXT and NLXT-RN+NPs groups. Consistent with the previous results, the effect of the NLXT-RN group was weakened more than NLXT. More critically, NLXT-NPs also had a significant regulatory impact on the content of the three indicators compared with the MCAO group (*p* < 0.05).

The above results indicate that NLXT can promote the activity of SOD and GSH-PX and inhibit the production of MDA; NLXT-NPs may be one of its pharmacological material bases for regulating oxidative stress.

### 2.6. NLXT-NPs Can Improve the Inhibition Effects of NLXT on Cell Apoptosis

**Figure 5:**
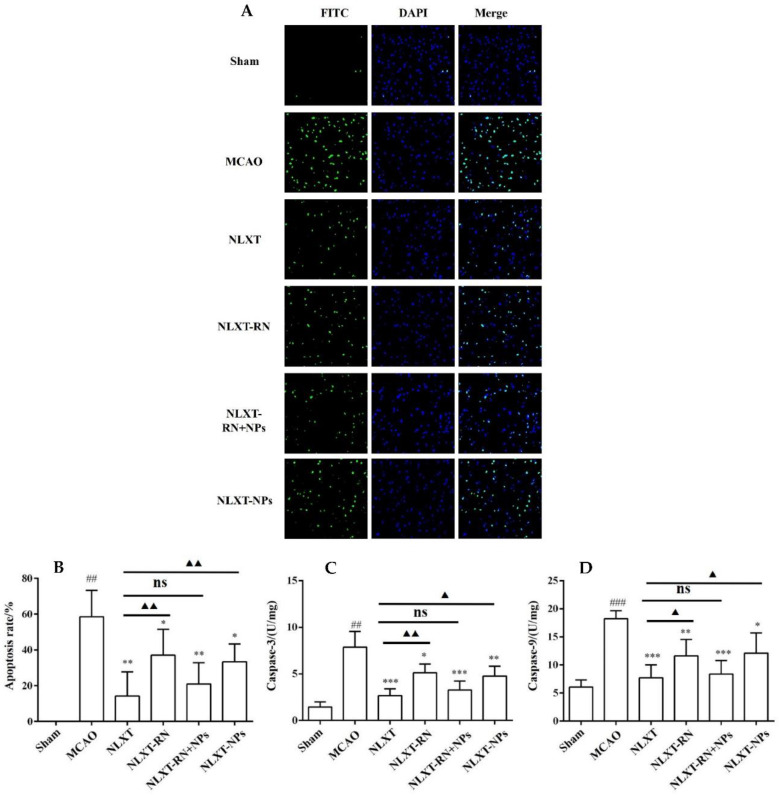
(**A**) The apoptosis of rat brain tissue after TUNEL staining was observed under 200-fold magnification (×200, *n* = 8). Effect of administration groups on the brain cells’ apoptosis rate (**B**) and caspase-3/9 (**C**,**D**) level of MCAO rat models (*n* = 8). ^##^ *p* < 0.01, ^###^ *p* < 0.001, compared with sham group; * *p* < 0.05, ** *p* < 0.01, *** *p* < 0.001; compared with MCAO group; ns: no significant difference, ^▲^ *p* < 0.05, ^▲▲^ *p* < 0.01, compared with NLXT group.

As shown in Figure 5, The cell apoptosis rate increased to (58.65 ± 14.60)% after modeling. In contrast, the apoptotic rate of NLXT (14.28 ± 13.54)% and NLXT-RN+NPs (21.00 ± 11.85)% groups were significantly decreased (*p* < 0.01). Furthermore, the apoptotic rate of NLXT-RN and NLXT-NPs were (37.09 ± 14.40)% and NLXT-RN+NPs (33.43 ± 9.95)%, respectively. Moreover, each administration group can reduce the activation of caspase-3/9 (*p* < 0.05) compared with the MCAO group. The NLXT group showed the most apparent inhibitory effect (*p* < 0.001), and there was no significant difference between NLXT and the NLXT-RN+NPs groups. In addition, compared with the NLXT group, the inhibitory effect of NLXT-RN was declined. These results showed that in addition to regulating oxidative stress, NLXT might also protect MCAO rats from brain damage by inhibiting abnormal neuronal apoptosis. And NLXT-NPs may affect its anti-apoptotic effect in various mechanisms.

### 2.7. Regulation Effect of NLXT on Bax/Bcl-2 Apoptosis Signal Pathway and the Influence of NLXT-NPs

**Figure 6:**
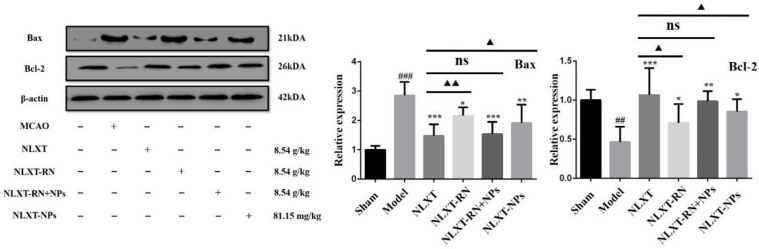
Effect of administration groups on the Bax/Bcl-2 protein expression level of MCAO rats (x ± s, *n* = 3). ^##^ *p* < 0.01, ^###^ *p* < 0.001, compared with sham group; * *p* < 0.05, ** *p* < 0.01, *** *p* < 0.001, compared with MCAO group; compared with MCAO group; ns: no significant difference, ^▲^ *p* < 0.05, ^▲▲^ *p* < 0.01, compared with NLXT group.

As shown in Figure 6, the western blot results showed that, compared with the MCAO group, the Bax protein expression level was significantly reduced after NLXT intervention (*p* < 0.001), and Bcl-2 was significantly increased (*p* < 0.01). Treatment with NLXT and NLXT-RN+NPs was associated with greater Bcl-2 and attenuated Bax expression, but the effect had an inevitable decline after removing nanoparticles. Consistent with the above results, NLXT-NPs can also reduce the expression of Bax and promote Bcl-2.

## 3. Discussion

### 3.1. Self-Assembly Mechanism of NLXT-NPs

Polysaccharide, one of the primary active ingredients in the TCM, showed high anti-oxidant and immune activity [30]. As a hydrophilic polymer, when we introduce hydrophobic groups into polysaccharides, it can self-assemble into nanoparticles under the influence of system hydrophobic interaction and temperature [20]. The hydrophilic polysaccharide chain exists outside the nano-particle, and the hydrophobic groups are wrapped in the inner core. The system temperature is one of the main factors affecting the morphology of nanoparticles, which is closely related to the particle size and shape [31,32]. In the process of NLXT decoction, polysaccharides and proteins gradually precipitate out and recognize each other to form glycoprotein structures [33]. Then, under thermal induction and hydrophobic action, the hydrophilic polysaccharide and the protein hydrophobic group self-assemble into a nanoparticle structure, the hydrophilic polysaccharide constitutes the shell of the nanoparticle, and the hydrophobic group of the protein constitutes the core [34]. During the self-assembly process, small-molecule active ingredients in the decoction can be adsorbed on the surface of nanoparticles or be encased inside through electrostatic adsorption, Van-Der-Waals force, π-π bonds, or other non-covalent interactions [19]. The surfactant has strong amphiphilicity, and it can be adsorbed on the surface of nanoparticles during the preparation process, which plays an essential role in maintaining the structural stability of nanoparticles [35]. Saponins have natural surface activity and are widely used as natural surfactants [36]. In this study, we have proved that some saponins were also detected; their molecular weights were all less than 3500 Da, and they can theoretically pass through the dialysis bag and be dialyzed out. Therefore, we speculated that during self-assembly, saponins adsorbed on the surface of nanoparticles by non-covalent means to maintain the structural stability of NLXT-NPs. Furthermore, similar to glycyrrhizin, saponins also have the potential to self-assemble into micelle particles. However, in this study, we can’t observe the existence of micelle particles under TEM and SEM, which further indicates that the saponins exist in the form of binding to glycoprotein nanoparticles.

### 3.2. Influence of Dialysis Conditions on the Stability of NLXT-NPs

The dialysis method is widely used to separate and purify nanoparticle solutions because of its mild reaction conditions, and it can prevent the extrusion of particles caused by external force [37]. However, Prusky [38] and Li [39] separated the green tea nanoparticles with different dialysis times, causing a significantly different result. The catechins and alkaloids in green tea nanoparticles can only be detected with dialysis less than 24 h in duration. It concluded that long-term pure water dialysis could result in the release of components loaded in nanoparticles. Therefore, in the separation of NLXT-NPs, the nanoparticle solution system was gradually purified but the saponins adsorbed on the particle surface may also slowly precipitate. When the saponins were not sufficient to maintain the stability of the nanoparticles, the structure of the NLXT-NPs gradually dissociated, which may explain the significant differences in morphology and composition between the NLXT-NPs dialyzed for 10 h and 12 h.

### 3.3. NLXT-NPs Can Improve the Anti-Oxidant Activity of NLXT

The occurrence of ischemic stroke can lead to an imbalance of the inherent anti-oxidant capacity of brain tissue, which in turn causes secondary reactions such as lipid peroxidation and inflammation. MDA is a product of lipid peroxidation. Its content reflects the level of oxygen free radicals in the body and the degree of lipid peroxidation. SOD can scavenge oxygen free radicals in the body, inhibit the formation of peroxides, and play an essential role in anti-oxidative stress damage. GSH-Px can promote the decomposition of H_2_O_2_, thereby maintaining the integrity of cell membrane structure and function. The activity of SOD, GSH-Px, and MDA levels are vital signs that reflect the level of oxidative stress in the body [40,41]. In this study, we compared the effects of each administration group on MCAO-induced oxidative stress. The NLXT-RN group was established as a negative control to investigate the influence of removing nanoparticles on the anti-oxidation efficacy of NLXT. Furthermore, to exclude the removal of non-nanoparticle components caused by dialysis, we established the NLXT-RN+NPs group to verify that the addition of NLXT-NPs can improve the anti-oxidation efficacy of NLXT-RN. Our result showed that NLXT-NPs could play an essential role in the anti-oxidant stress effect of NLXT, which did not exceed our expectations. The composition of NLXT-NPs, including polysaccharides and saponins, are all bioactive ingredients for regulating nerve function recovery and improving microcirculation in the central nervous system. These ingredients may be the material basis for NLXT-NPs to anti-oxidative stress damage.

### 3.4. NLXT-NPs Can Improve the Inhibition Effects of NLXT on Cell Apoptosis

Apoptosis refers to programmed cell death regulated by multiple genes, which involves Caspase activation, mitochondrial transmembrane potential drop, phosphatidylserine eversion, and regular DNA breaks [42,43]. It is important for the body to maintain homeostasis under physiological conditions. After ischemic stroke, the body will further induce oxidative stress and inflammatory hierarchical response due to ischemia and hypoxia, which will cause the pathological apoptosis of brain tissue cells and aggravate brain damage [44]. Therefore, it is imperative to inhibit pathological cell apoptosis to prevent and treat ischemic stroke. It has been reported that the Caspase-3/9 signaling pathway serves as a potent effector in promoting cell death and apoptosis. Caspase-3, the primary executor of apoptosis, can cause apoptosis by specifically lysing its substrate; Caspase-9 can activate Caspase-3 to initiate the Caspase cascade reaction [45]. Besides, the Tunel method is one of the commonly used methods to detect apoptosis. Our research combines the enzyme activity of Caspase-3/9 and Tunel staining to evaluate the apoptosis level of each group. Moreover, Bcl-2 and Bax are two classic anti-apoptotic and pro-apoptotic proteins that can regulate cell apoptosis by forming homodimers or heterodimers. Bax and Bcl-2 directly determine the permeability of various channels in the outer mitochondrial membrane through the formation of homologues or heterodimers, thereby determining the survival of cells [46]. The above experimental results show that NLXT and NLXT-NPs can reduce the level of apoptosis; their mechanisms were all related to the regulation of Caspase3/9 and Bax/Bcl-2. Notably, removing NLXT-NPs can significantly decrease the anti-apoptotic effect of NLXT. Therefore, NLXT-NPs can be regarded as one of the pharmacodynamic material bases for NLXT to regulate the Caspase3/9 and Bax/Bcl-2 pathways. The results of pharmacological experiments showed that research on the TCM pharmacodynamic material basis needs to consider the direction of nanoparticles.

## 4. Materials and Methods

### 4.1. Materials

NLXT consists of six herbs and one animal material. The crude slices of Astragali radix (No: 200,601), Chuanxiong Rhizoma (No: 200,601), Notoginseng radix et rhizome (No: 200,601), Gastrodiae rhizome (No: 200,601), Scolopendra (No: 160,801), Angelicae Sinensis radix (No: 200,601) and Carthami Flos (No: 200,601) were purchased from herb companies in Bozhou (Anhui). The primary antibodies of Bcl-2 (cat. no. bsm-33047M), and Bax (cat. no. bsm-33283M) were purchased from Bioss (Beijing, China). Elisa Kit of SOD (cat. no. A001-3-2), MDA (cat. no. A003-1-2), GSH-Px (cat. no. A005-1-2), Caspase-3 (cat. no. G015-1-3), Caspase-9 (cat. no. G018-1-3), and Tunel apoptosis detection kit (cat. no. G002-1-2) were purchased from Nanjing Jiancheng Bioengineering Institute (Nanjing, China).

### 4.2. Preparation of NLXT

Firstly, the herbs of NLXT were weighed in proportion (Astragali radix: Chuanxiong rhizome: Notoginseng radix et rhizome: Gastrodiae rhizome: Chilopoda Scolopendra: Carthami Flos: Angelicae Sinensis Radix = 15:5:3:5:2:5:5) and soaked for thirty minutes. The mixture was boiled twice with 10- and eightfold volume deionized water for an hour. Next, the first decoction was merged with the second decoction, and the powdered medicine of scolopendra was immediately added to the decoction.

### 4.3. Isolation of NLXT-NPs

**Figure 7:**
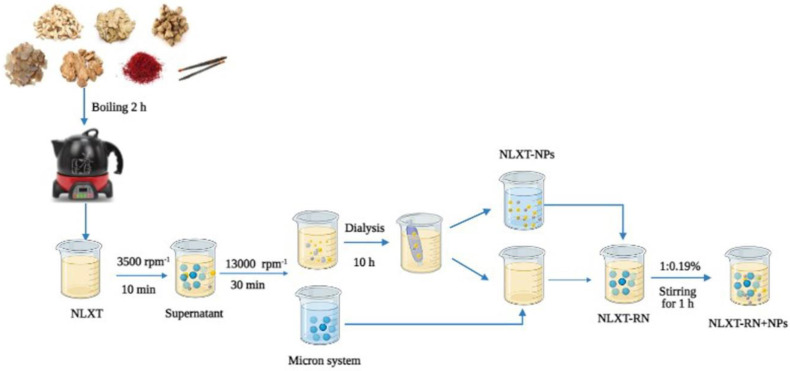
NLXT−NPs isolation process diagram.

After preparation, NLXT was centrifuged at 3500 rpm for 10 min to remove the dregs, then rotary evaporated and concentrated to 1.0 g·mL^−1^. The supernatant was filtered and then centrifuged at 13,000 rpm·min^−1^ for 30 min to remove micron particles. Additionally, 40 mL of the supernatant was added it to 1/2 of the dialysis bag (3500 Da), put the dialysis bag into a beaker with 2000 mL deionized water, and dialyzed for 10 h in a water bath magnetic stirrer at 600 r·min^−1^. We collected and replaced the external dialysis fluid every 2 h. Finally, the dialysate was freeze-dried to obtain the freeze-dried powder of NLXT-NPs. The dialysis external fluids were combined and concentrated by rotary evaporation to 40 mL; after being mixed with micron particles, the NLXT-RN solution was then obtained. Then, according to the yield of NLXT-NPs (0.19% ± 0.01%), 76.0 mg of NLXT-NPs freeze-dried powder was added to the NLXT-RN solution after magnetic stirring at 37 °C in a water bath for1 h, the NLXT-RN+NPs solution was obtained (Figure 7).

### 4.4. Size Distribution, Surface Charge, and Morphology of NLXT-NPs

The size distribution and surface charge of NLXT-NPs were measured using a laser particle analyzer (ZEN 3690 Laser Scatter particle analyzer, Malvern Panalytical, UK). The morphology of the NLXT-NPs was observed by transmission electron microscopy (TEM, HT7700, Hitachi, Tokyo, Japan) and scanning electron microscope (SEM, Regulus8100, Hitachi, Tokyo, Japan). In addition, Fourier infrared spectroscopy was used to analyze the structural characteristics of NLXT-NPs.

### 4.5. Chromatography and Mass Spectrometry Conditions

The chromatography separation was performed on a Waters AcquityTM UPLC system (Waters Corporation, MI, USA). The analysis of samples was carried out on a Waters ACQUITY-CSH-C18 (2.1 mm × 100 mm, 1.7 μm) column with a gradient elution using 0.1% formic acid (solvent A) and acetonitrile (solvent B). The gradient elution was set as follows: 0–5 min, 10–15% B; 5–15 min, 15–20% B; 15–30 min, 20–30% B; 30–40 min, 30–40% B; 40–45 min, 40–60% B; 45–47 min, 60–10% B. In addition, the flow rate, column temperature and injection volume were set to 0.2 mL/min, 30 °C and 2 μL, respectively.

A Waters Xevo G2 Q/TOF mass spectrometer (Waters Corporation, Milford, CT, USA) equipped with an ESI source was used for mass spectrometric detection. All samples were detected in negative mode. The complete scan data were acquired from 50 to 1200 Da, using a capillary voltage of −2.5 kV for negative ion mode, sampling cone voltage of 50 V for negative ion mode, extraction cone voltage of 4.0 V, source temperature of 110 °C (ESI−), cone gas flow of 50 L/h, desolvation gas (N2) flow of 600 L/h and desolvation gas temperature of 350 °C. The collision voltage was set as 6.0 eV for low-energy scan and 20–80 eV for the high-energy scan. To ensure the mass accuracy and spectral reproducibility of the MS condition, leucineenk ephalin ([M-H] − (*m*/*z* 554.2615) in negative ion mode), consisting of a 200 pg/mL solution, was set as an external reference (Lock-SprayTM) at a flow rate of 10 µL/min via a lockspray interface.

### 4.6. Preparation of Sample Solutions

NLXT-NPs was dissolved into methanol solution. It was then ultrasonically dispersed for 2 h to completely release the small molecule ingredients contained in the nanoparticles, and then centrifuged at 13,000 r·min^−1^ for 10 min to remove the glycoprotein precipitate. Before UPLC-Q-TOF-MS analysis, the supernatant was filtered through a 0.22 µm filter membrane.

### 4.7. Animals and MCAO Model Construction

Male Sprague-Dawley rats (220~240 g) were purchased from the Experimental Animal Center of Anhui Province. All animals were kept in a pathogen-free environment and fed ad-lib. The procedures for care and use of animals were approved by the Ethics Committee of the Anhui University of Chinese Medicine, Anhui (AHUCM-rat-2020051), China and all applicable institutional and governmental regulations concerning the ethical use of animals were followed. In our research, 48 rats were randomly divided into six groups, respectively, the sham group, the MCAO model group, the NLXT group (8.54 g/kg), the NLXT-RN group (8.54 g/kg), the NLXT-RN+NPs group (8.54 g/kg), and the NLXT-NPs group (81.15 mg/kg). The MCAO model construction method is as follows (28): rats were anesthetized with 3% sodium pentobarbital intraperitoneally (1 mL/100 g) and maintained at core temperature by placement on a heating pad, and then the neck was sliced in the exact center, separating a left side common carotid artery, external carotid artery and internal carotid artery a ligature was placed in the carotid artery near the heart and at the branch of the external carotid artery. It was sliced to shear 1 in the carotid artery branch to have a pure paint of diameter for 0.20 mm, fishing thread carries to insert toward internal carotid artery, continuing to push forward until a slight resistance was felt, then about an additional after 2 h, the fishing thread was slowly promoted, and then reperfusion was implemented. In addition, to remove the influence of surgical factors on the experimental results, the sham operation group was set as the reference of the MCAO model. In the sham operation group, we only performed anesthesia and vascular separation, and no fishing thread was used in the middle artery. Compared with the normal control, the sham operation group can more reasonably reflect the effects of cerebral ischemia on various biochemical indicators in rats, making the experimental results more convincing. After modeling, rats were scored according to the Garcia JH scoring rules, and scores of 1 to 2 were selected for follow-up experiments. Each administration group was given intragastric administration twice a day for seven days. The sham and the MCAO model groups were given normal saline (1 mL/100 g).

### 4.8. Evaluation of Neurological Function Score (NFS) and Infarct Volume

After administration, rats were scored again according to their autonomous movement, symmetry of posture, forelimb extension, screening experiment, tactile reflex on both sides of the body, and tactile reflex on both sides of the beard. The rats were then anesthetized and sacrificed, and the brains were quickly removed and frozen at −20 °C for 30 min before slicing. The slices were stained with 2% TTC (RS4130, G- clone, Beijing, China) for 30 min at 37 °C in the dark. After being fixed with 4% paraformaldehyde at 4 °C for 24 h, photos of each slice were taken, and the infarct volume was calculated by Image J software.

### 4.9. Histopathological Changes in Cerebral Cortex

After being dehydrated, embedded in paraffin and sectioned, the rat brain tissues were stained with hematoxylin for 5 min and 1% water-soluble eosin staining solution for 2 min, then pictures were taken under the 200× field of view of the inverted microscope.

### 4.10. Oxidative Stress Level Measurement

The activity of superoxide dismutase (SOD), glutathione peroxidase (GSH-Px), and the level of malondialdehyde (MDA) were determined using the corresponding kits, according to the manufacturers’ instructions. Subsequently, the absorbance was determined at 450 nm (SOD), 532 nm (MDA), or 422 nm (GSH-Px) using a microplate reader (Thermo Fisher, Multiskan Spectrum, Fremont, CA, USA).

### 4.11. Cell Apoptosis Measurement

After intracardiac perfusion was performed as described above, the brains were removed, fixed, dehydrated, and sectioned. The sections were then subjected to TUNEL staining according to the operation manual. Furthermore, the level of Caspase-3/9 was also measured by the corresponding kits.

### 4.12. Western Blot Analysis

Approximately 100 mg of cortical tissue from the infarcted area was taken, rapidly ground with liquid nitrogen, and added to 900 μL of RIPA (Servicebio, Wuhan, China). A Lysis Buffer contained the protease inhibitor PMSF in a 100:1 ratio of RIPA to PMSF. After lysis on ice for one hour, the mixture was centrifuged at 12,000 rpm for 10 min at 4 °C. The supernatant was then collected and added with 1/4 volume of the loading buffer. After being heated at 100 °C for 10 min to denature the protein, the mixture was cooled, divided, and frozen for use. Protein concentrations were determined using a sensitive BCA protein assay kit (Servicebio, Wuhan, China). The protein samples (40 µg/lane) were then separated by 10% SDS-PAGE and transferred onto a PVDF membrane ((Servicebio, Wuhan, China)). Subsequently, the membrane was blocked with 5% skimmed milk for 2 h at room temperature, washed with Tris-buffered saline containing 0.2% Tween-20 (TBST; Servicebio, Wuhan, China), and incubated with the primary antibodies at 4 °C overnight. After washing with TBST, the membrane was incubated with a goat anti-rabbit horseradish peroxidase (HRP)-conjugated secondary antibody (1:3000) at room temperature for 2 h. Finally, protein bands were visualized using an ECL Chemiluminescence Kit (Servicebio, Wuhan, China) on an ultra-sensitive multi-function imager (Amersham Image 600). The proteins bands were quantified using the ImageJ software. The gray value of the target protein was normalized to that of β-actin (Bioss, Beijing, cat. no. bsm-33036M).

### 4.13. Statistical Analysis

Each experiment was separately performed at least three times. The data were expressed as the mean ± standard deviation (SD). A one-way analysis of variance (ANOVA) with post hoc Bonferroni was performed. Differences were considered significant at *p* < 0.05. Statistical analysis was performed using SPSS 23.

## 5. Conclusions

In this study, we used high-speed centrifugation combined with dialysis to separate the na-noparticles in NLXT. Bioactive compounds such as Ginsenoside Rg1, Ginsenoside Rb1, and Astragaloside IV were demonstrated to be carried by the NLXT-NPs, which may have a profound impact on their in vivo transport, bioavailability and therapeutic effects. In addition, our research also found that the neural protection effect of NLXT was closely related to these nanoparticles; the removal of NLXT-NPs can significantly reduce its oxidative stress protection and anti-apoptotic effects. Our research provides a novel addition TCM compatibility theory in that the nanoparticles isolated from decoction play an essential role in the efficacy of compound prescriptions.

## Figures and Tables

**Table 1:** Neurological scores and cerebral infarction volumes of rats (x ± s, *n* ≥ 6).

Group	NFS/Score	Infarction Volume/%
Sham	17.17 ± 0.98	0.00 ± 0.00
MCAO	6.67 ± 0.82 ^#^	47.83 ± 5.31 ^#^
NLXT	13.17 ± 1.17 *	19.00 ± 3.35 *
NLXT-RN	10.17 ± 1.17 *^▲^	27.50 ± 5.01 *^▲^
NLXT-RN+NPs	12.83 ± 0.75 *	22.67 ± 4.41 *
NLXT-NPs	9.33 ± 1.86 *^▲^	31.17 ± 3.97 *^▲^

**Note:** Compared with the sham group, ^#^ *p* < 0.05; Compared with MCAO group, * *p* < 0.05, compared with MCAO group; ^▲^ *p* < 0.05, compared with NLXT group.

## Data Availability

The data presented in this study are available in this article.

## Sample Availability

Samples of the compounds are available from the authors.
